# Strategies to improve the regulatory assessment of developmental neurotoxicity (DNT) using *in vitro* methods

**DOI:** 10.1016/j.taap.2018.02.008

**Published:** 2018-09-01

**Authors:** Anna Bal-Price, Francesca Pistollato, Magdalini Sachana, Stephanie K. Bopp, Sharon Munn, Andrew Worth

**Affiliations:** a European Commission, Joint Research Centre (JRC), Ispra, Italy; b Organisation for Economic Co-operation and Development (OECD), 2 rue André Pascal, 75775 Paris, Cedex 16, France

**Keywords:** Developmental neurotoxicity, Human *in vitro* test systems, Adverse outcome pathways, Integrated Approaches to Testing and Assessment, Regulatory purposes

## Abstract

Currently, the identification of chemicals that have the potential to induce developmental neurotoxicity (DNT) is based on animal testing. Since at the regulatory level, systematic testing of DNT is not a standard requirement within the EU or USA chemical legislation safety assessment, DNT testing is only performed in higher tiered testing triggered based on chemical structure activity relationships or evidence of neurotoxicity in systemic acute or repeated dose toxicity studies. However, these triggers are rarely used and, in addition, do not always serve as reliable indicators of DNT, as they are generally based on observations in adult rodents. Therefore, there is a pressing need for developing alternative methodologies that can reliably support identification of DNT triggers, and more rapidly and cost-effectively support the identification and characterization of chemicals with DNT potential.

We propose to incorporate mechanistic knowledge and data derived from *in vitro* studies to support various regulatory applications including: (a) the identification of potential DNT triggers, (b) initial chemical screening and prioritization, (c) hazard identification and characterization, (d) chemical biological grouping, and (e) assessment of exposure to chemical mixtures. Ideally, currently available cellular neuronal/glial models derived from human induced pluripotent stem cells (hiPSCs) should be used as they allow evaluation of chemical impacts on key neurodevelopmental processes, by reproducing different windows of exposure during human brain development. A battery of DNT *in vitro* test methods derived from hiPSCs could generate valuable mechanistic data, speeding up the evaluation of thousands of compounds present in industrial, agricultural and consumer products that lack safety data on DNT potential.

## Highlights

• Current *in vivo* developmental neurotoxicity (DNT) testing is not efficient and coverage is sparse.
• *In vitro* mechanistic data could support various regulatory applications.
• Human induced pluripotent stem cell-derived neuronal models are recommended for DNT testing.
• Further development of adverse outcome pathways relevant to DNT is urgently needed.
• *In vitro* approaches should be included in regulatory DNT testing

## 1 Introduction

The developing nervous system is known to be more vulnerable to chemical exposure as compared to the adult nervous system (Spyker, 1975; NRC, 1993; Rodier, 1995; Grandjean and Landrigan, 2006). The higher vulnerability of the developing brain results from the complex, specific developmental processes, such as the commitment and differentiation of neural progenitor cells followed by glial and neuronal cell proliferation, migration, differentiation into various neuronal and glial subtypes, synaptogenesis, pruning, myelination, networking and terminal functional neuronal and glial maturation (Rice and Barone Jr, 2000; Hogberg et al., 2009, 2010; Stiles and Jernigan, 2010; Krug et al., 2013; Yang et al., 2014). A challenge in the evaluation of developmental neurotoxicity (DNT) induced by an exogenous chemical is that the neurodevelopmental outcome depends not only on the kind of exposure (dose, duration), but also on the developmental stage of the brain at the time of exposure (Rice and Barone Jr, 2000). Additionally, the immature blood brain barrier (BBB) is not completely formed at least until 6 months after birth (Rodier, 1995) thus facilitating the entrance of a chemical into the foetal/neonatal brain (Adinolfi, 1985).

Despite the recognized need for a more systematic and rigorous evaluation of DNT at the regulatory level (Bal-Price et al., 2012, 2015a), DNT evaluation is not a mandatory requirement in the USA or the European Union for pesticides, biocides, pharmaceuticals or industrial chemicals, and it is performed only as higher tiered tests that are triggered based on structure activity relationships or evidence of neurotoxicity observed in standard *in vivo* adult, developmental or reproduction studies (Makris et al., 2009; Bal-Price et al., 2010, 2012), either after acute exposure (*e.g.*, OECD TGs 402 (OECD, 2017b), 403 (OECD, 2009a), 420 (OECD, 2002a), 423 (OECD, 2002b), 436 (OECD, 2009b) and 425 (OECD, 2008b)), or repeated dose treatment, sub-acute TG 407 ((OECD, 2008a) and sub-chronic TG 408 (OECD, 1998) or chronic exposure (OECD TG 452 (OECD, 2009c)). At the same time, recent societal concerns have been raised linking the increase in children's neurodevelopmental impairments (*e.g.*, learning disabilities, autism, attention deficit hyperactivity disorder (ADHD)) to chemical exposures (Bennett et al., 2016; Fritsche et al., 2017; Grandjean et al., 2017).

For regulatory purposes, the identification of chemicals with DNT potential is primarily based on the OECD TG 426, which is an update to the US EPA DNT Guideline (OPPTS 8706300, EPA 712-C-98-239 (US-EPA, 1998)) and the OECD TG 443 – extended one-generation reproductive toxicity study (OECD, 2011). These TGs are entirely based on animal studies since there are still no regulatory accepted alternative methods for this endpoint. TG 426 and 443 require neurobehavioral determination of cognitive, sensory and motor functions accompanied by morphometric and histopathological evaluation of the brain. Additional testing specifically of offspring that have been exposed *in utero* and during early lactation includes also sexual maturation evaluation (OECD TG 426 and OECD TG 443) (OECD, 2007, 2011), assessments of behavioral ontogeny and learning and memory testing (OECD TG 426) (OECD, 2007). However, OECD TG 426 is rarely performed as it is very resource intensive in terms of animals, time and overall cost (Rovida and Hartung, 2009; Tsuji and Crofton, 2012), and has been used only for a limited number of pesticides and industrial chemicals (approximately 120) (Crofton et al., 2012; Kadereit et al., 2012; van Thriel et al., 2012). Therefore, there is only a small amount of DNT studies available, mainly for pesticides (Grandjean and Landrigan, 2006; Bjorling-Poulsen et al., 2008), that have contributed to risk assessments and regulatory decision making (Makris et al., 2009). This highlights the pressing need to develop alternative approaches as part of a testing strategy that can identify at least DNT alerts and guide chemical prioritization for further testing at a lower tier level in a more rapid and cost-effective manner.

Decades of *in vitro* work using rodent and human neuronal and glial cellular models have delivered a range of reliable *in vitro* assays and data that permit quantitative evaluation (*via* concentration-response relationships) of the impact of a compound on various stages of brain development. These *in vitro* DNT assays once assembled in a battery of tests could benefit by the inclusion of information derived from *in silico* approaches (*e.g.*, QSAR) and non-mammalian animal models (*e.g.*, zebrafish, medaka or *C. elegans*), if required, for neuro-behavioral endpoints. The gathering of data from multiple information sources, primarily coming from the battery of the *in vitro* DNT test methods, combined with available *in vivo* and epidemiological human data, could be used to develop Integrated Approaches to Testing and Assessment (IATA) designed in a fit-for-purpose manner in relation to different regulatory purposes (chemical screening for further prioritization, hazard identification/characterization or risk assessment).

The battery of the *in vitro* DNT test methods included in an IATA should be preferably based on human derived *in vitro* models due to species differences in chemical effects on neurodevelopmental key events (Baumann et al., 2016). Relevant DNT endpoints should be anchored to key neurodevelopmental processes and pathways critical for brain development. In this regard, EFSA has published a detailed report on the evaluation of the currently available *in vitro* test methods, including human models, as well as other alternative approaches (*in silico* modeling, read-across, non-mammalian models, *etc.*) suitable for DNT testing (Fritsche et al., 2015), concluding that a variety of *in vitro* methods covering early and late stages of neurodevelopment are already available and could be used to predict DNT effects.

This report describes how such IATA, depending on the problem formulation, could support (a) initial chemical screening and prioritization of chemicals based on their potential to induce DNT, (b) hazard identification and characterization for specific chemical risk assessment, (c) grouping of chemicals according to their DNT properties, (d) assessment of combined effects following exposure to multiple chemicals (mixture risk assessment), and (e) identification of potential triggers for DNT testing.

For these different regulatory purposes, human cell-based systems are strongly recommended as the most relevant to reduce the uncertainty in extrapolation of results and to improve prediction of human toxicity (NRC, 2007).

## 2 Human *in vitro* test systems (models and endpoints) for human DNT evaluation

**Fig. 1:**
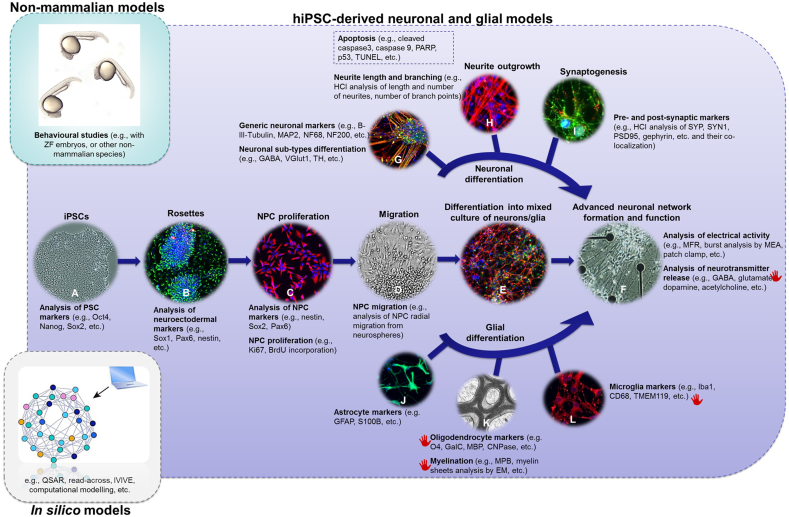
Battery of *in vitro* assays anchored to key neurodevelopmental processes, non-mammalian models and *in silico* approaches suitable for evaluation of DNT effects. Human induced pluripotent stem cells (hiPSCs) (A) can be used to form rosettes (neuroectodermal cells, resembling neural tube formation *in vitro*) (B); neural progenitor cells (NPCs) (C) can be derived from rosettes, their migration can be measured (D), and NPCs can be further differentiated into various neuronal and glial sub-types (E–L). Apart from the image showing myelination (K) (modified from https://www.mpg.de/11583034/original-1508156154.jpg), the displayed images are representative pictures of IMR90-hiPSCs differentiated in house as detailed in (Pistollato et al., 2017b). These key neurodevelopmental processes can be measured by gene and protein analysis of markers specific for PSCs, neuroectoderm, and NPCs, and sequential neurodevelopmental processes as shown in the figure. Such analysis can be combined with functional *in vitro* assays (*e.g.* MEA measurments) and non-mammalian behavioral studies, if required, (*e.g.*, ZF embryos) and/or *in silico* models (*e.g.*, QSAR, read-across, IVIVE, *etc.*) in a battery of tests to support DNT testing. Further efforts are still needed to optimize the assays for evaluation of chemical impact on hiPSCs and NPCs differentiation into mature oligodendrocytes (able to form myelin), microglia, and fully mature neurons (indicated by a red hand symbol). Images show staining for: nestin (green)/β-III-tubulin (red) (B), nestin (red) (C), GFAP (green)/β-III-tubulin (red) (E), synapsin-1 (green)/β-III-tubulin (red) (G), β-III-tubulin (red) (H), synaptophysin (green)/PSD95 (red) (I), GFAP (green) (J), and Iba1 (red) (L). Other abbreviations: Oct4, octamer-binding transcription factor 4; Sox1 (and Sox2), Sex Determining Region Y-Box 1 (and Box 2); Pax6, paired Box 6; HCI, high content imaging; PARP, poly (ADP-ribose) polymerase; MAP2, microtubule-associated protein 2; NF68, neurofilament 68 kDa; NF200, neurofilament 200 kDa; GABA, gamma-aminobutyric acid; VGlut1, vesicular glutamate transporter 1; TH, tyrosine hydroxylase; GFAP, glial fibrillary acidic protein; S100B, S100 calcium-binding protein B; O4, oligodendrocyte marker 4; GalC, galactocerebroside; MBP, myelin basic protein; CNPase, 2′,3′-cyclic-nucleotide 3′-phosphodiesterase; Iba1, ionized calcium binding adaptor molecule 1; CD68, cluster of differentiation 68; TMEM119, transmembrane protein 119; SYP, synaptophysin; SYN1, synapsin 1; PSD95, postsynaptic density protein 95; EM, electro-microscopy; MFR, mean firing rate; MEA, multi-electrode array; IVIVE, *in vitro* to *in vivo* extrapolation; ZF, Zebrafish; TUNEL, Terminal deoxynucleotidyl transferase dUTP nick end labelling. (For interpretation of the references to colour in this figure legend, the reader is referred to the web version of this article.)

In the last decades, several cell culture systems derived from different species (mainly human and rodent) have been used for *in vitro* DNT testing (Costa, 1998; Harry et al., 1998; Coecke et al., 2007; Bal-Price et al., 2008, 2010; Gassmann et al., 2010). With regard to the human models suitable for DNT evaluation, various neuroblastoma cell lines and stem cell-derived systems are available (Bal-Price et al., 2012). However, transformed/immortalized cell lines present some disadvantages, as the expression of tumor growth-related genes may affect cell response upon chemical exposure. Alternatively, human *in vitro* neuronal cultures derived from neural progenitor cells (NPCs) have been intensively studied over the past decade as they are self-renewable, although not immortalized, and can be differentiated into several neuronal and glial cell types (Moors et al., 2007; Breier et al., 2010; Pistollato et al., 2014, 2017b). For instance, human primary NPCs derived from brain fetal tissues and grown as neurospheres can be used to mimic *in vitro* critical brain developmental processes, including proliferation, apoptosis, migration and differentiation (Fritsche et al., 2011), and are therefore considered as the most suitable for DNT testing (Fritsche et al., 2005; Schmuck et al., 2017). NPCs can be obtained from two major types of pluripotent stem cells (PSCs), human embryonic (hESCs) and human induced pluripotent stem cells (hiPSCs). Taking into consideration the ethical issues and the differences in national legislation regulating the generation and use of hESCs and/or fetal-derived tissues, hiPSC-derived neuronal and glial models are currently gaining increasing scientific interest for their applicability in a broad range of *in vitro* pharmacological and toxicological studies, including DNT. HiPSCs can be expanded in culture in an undifferentiated state and then differentiated into most cell types (*e.g.*, cardiomyocytes, hepatocytes, muscle cells, *etc.*) including neurons, allowing to quantify *in vitro* tissue-specific biological processes in a high-throughput manner (Scott et al., 2013). HiPSC-derived mixed cultures of neuronal and glial cells (Fig. 1), are considered particularly suitable for DNT, rather than for adult neurotoxicity evaluation (Hofrichter et al., 2017; Pistollato et al., 2014, 2017b), since these cells (and hESCs) do not reach a terminal level of differentiation and function characteristic for adult brain physiology (Yla-Outinen et al., 2010), even after long term culture (Amin et al., 2016). Assessing hiPSC-derivatives similarity to primary tissue is of crucial importance to verify the level of concordance between data obtained from hiPSC-derived neuronal/glial models and those obtained with primary cell systems. In this regard, Hofrichter and co-workers have recently compared the neuronal and astrocytic differentiation capacity of hiPSC-derived NPCs with that of primary human NPCs (Hofrichter et al., 2017). While primary NPCs can be easily differentiated into nestin^+^ and/or glial fibrillary acidic protein (GFAP)^+^ cells, hiPSC-derived NPCs tend to first differentiate into β-III-tubulin^+^ cells, which suggests an earlier neurodevelopmental phenotype of this cell model. Interestingly, migration of hiPSC-NPCs and primary NPCs was similarly impacted by methylmercury chloride treatments, indicating that hiPSC-derived NPCs can be suitable to model cell migration *in vitro* (Hofrichter et al., 2017).

One possible limitation of hiPSC-derived models is that the amount of glial cells is generally low in comparison with the *in vivo* developing brain tissue. Glial cells including oligodendrocytes (responsible for myelin formation), microglia (involved in inflammatory response) and astrocytes (presenting anti-oxidant capacity, and mediating release of pro-survival factors, uptake of glutamate, ion balance, *etc.*) play a critical role in chemically-induced mechanisms of neurotoxicity (Aschner et al., 2005). Several protocols have been developed to optimize the differentiation of astrocytes from PSCs, and specifically from hiPSCs (Emdad et al., 2012; Chandrasekaran et al., 2016) resulting in a higher yield of this type of glial cells. The optimization of culturing protocols based on the use of defined factors and co-culture with astrocytes has allowed the generation of microglia-like cells from hiPSCs, showing the phenotypic and gene expression profiles and functional properties similar to those of brain-derived microglia (Pandya et al., 2017).

Additionally, three dimensional (3D) culture systems have shown promise in better recapitulating *in vitro* brain tissue physiology and microenvironmental conditions, yielding higher levels of oligodendroglia differentiation and myelination, and allowing to investigate *in vitro* neuron-glia interactions and functions (Pamies et al., 2017b). Lancaster and colleagues reached an even higher level of biological complexity, by developing a human PSC-derived 3D organoid culture system, obtained first by embedding pre-formed neuroepithelial tissues into droplets of Matrigel in a stationary phase, and second by transferring such tissue droplets into a spinning bioreactor in the presence of differentiation medium. This approach allowed obtaining cerebral organoids able to recapitulate various discrete but also inter-dependent brain regions, similar to the *in vivo* 3D cytoarchitecture of cerebral cortex-like structures characterized by the presence of progenitor cells, radial glial stem cells, mature neuronal and glial subtypes (Lancaster et al., 2013). HiPSCs have been also recently differentiated into brain microvascular endothelial cells suitable to mimic the functionality of the BBB *in vitro* (Canfield et al., 2017; Hollmann et al., 2017).

Moreover, many of the neurodevelopmental signaling pathways that are deregulated in brain disorders (*e.g.*, Notch, mTOR, GSK3B, Stat3, FoxO, BDNF, ERK, CREB, PI3K, AKT, MAPK, PDGFR-PLCγ1, Wnt, several miRNAs, *etc.*) (Imayoshi et al., 2013; Bengoa-Vergniory and Kypta, 2015; Hevner, 2015; Ehrlich and Josselyn, 2016; Kang et al., 2016), reviewed in (Fritsche, 2017), have been found to be expressed both at gene and protein level in hiPSC-NPCs and their neuronal derivatives (Pistollato et al., 2014). The identification of these pathways allows studying perturbations of physiological signaling *in vitro*, occurring as a consequence of chemical treatment (hereafter named as “toxicity pathways”). For instance, we have previously shown that chemical-induced inhibition of the cAMP responsive element binding protein (CREB) pathway in IMR90-hiPSC-derived neuronal and glial culture was associated with inhibition of neurite outgrowth and synaptogenesis, as well as MAP2^+^ neuronal cell decrease (Pistollato et al., 2014). More recently, we reported that rotenone-dependent activation of the Nrf2 signaling pathway, a master regulator of antioxidant response (Sporn and Liby, 2012), elicited astroglial cell reactivity and dopaminergic neuronal cell death in IMR90-hiPSC-derived NPCs further differentiated into neurons and glia (Pistollato et al., 2017a; Zagoura et al., 2017). Altogether these and other studies indicate that hiPSC-derived neuronal/glial cells are suitable models for studying chemically-induced DNT resulting from neurodevelopmental pathway perturbations.

It is important to add that the DNT community is striving for implementing tests for endocrine disruptors (ED) evolution into the DNT *in vitro* testing battery but, yet, clearly more work is needed for DNT effects triggered by ED (Dach et al. (2017).

It is important to stress that growing stem cells in a stable state and delivering reliable and well-characterized cultures for toxicity assessment require a high level of standardization of both undifferentiated and differentiated cell cultures, in order to ensure the establishment of robust test systems. It is therefore of pivotal importance to define and internationally agree on a set(s) of quality control parameters suitable to properly characterize stem cell-derived models before using them for toxicity testing (Coecke et al., 2005), especially those derived from PSCs (Pistollato et al., 2012; Pamies et al., 2017a).

Currently, robust human stem cell-based *in vitro* models are used to evaluate key neurodevelopmental processes, known to be specific for normal brain development and maturation. These include commitment and proliferation of neural stem cells, apoptosis, cell migration, neuronal and glial differentiation, neurite outgrowth, myelination, axonal and dendritic elongation, synapse formation, synapse pruning, neurotransmitter receptor profiling, development of neuronal connectivity, spontaneous electrical activity, *etc.* (Coecke et al., 2007; Fritsche et al., 2015). Most of these DNT-specific *in vivo* processes can now be recapitulated under *in vitro* conditions and quantitatively assessed upon an exposure to a chemical (see examples in Table 1) using a wide range of different *in vitro* models, including hiPSC-derived neuronal cultures as the most relevant to human DNT testing. For example, high-content image analyses were performed by the U.S. EPA to assess the effects on neurite outgrowth of approximately 300 chemicals (Mundy et al., 2010), using hiPSC-derived neuronal culture (for 80 chemicals) (Druwe et al., 2016; Ryan et al., 2016), to measure neural proliferation (Breier et al., 2008; Mundy et al., 2010), and synaptogenesis (Harrill et al., 2011). Neuronal network formation and function has been also investigated in different cell systems (Mundy et al., 2008), including hiPSC-derived neuronal models (*e.g.*, (Amin et al., 2016)), by measuring electrical activity using multi-electrode array (Hogberg et al., 2011; Novellino et al., 2011; Valdivia et al., 2014; Vassallo et al., 2017).

**Table 1:** Examples of the apical *in vivo* endpoints required by OECD TG 426 and TG 424 (adapted from (Aschner et al., 2017). Each of the *in vivo* endpoints could be linked to the perturbation of key cell biological processes (*e.g.*, altered apoptosis, cell migration or cell proliferation or differentiation may lead to size differences of brain regions). The changes of cellular biological processes may be modelled and studied by using *in vitro* assays applied to hiPSC-derived mixed culture of neuronal and glial cells.

Methods *in vivo*	Outcome	Cell biological processes
Gross morphology	Brain measures ↓↑ Brain parts missing Malformation	→Proliferation, apoptosis →Proliferation, differentiation →Proliferation, migration, differentiation
Histopathology	Necrosis Pyknosis Neuronal Degeneration Astrocytosis Layer thickness ↓↑	→Cytotoxicity →Apoptosis, necrosis →Neurotoxicity →Glial proliferation, GFAP content →Proliferation, migration, myelination, cell death
Morphometry	Layer thickness ↓↑ Morphology	→Proliferation, migration, myelination →Proliferation, migration, differentiation
Learning/memory/motor activity	↓↑	→Synaptogenesis →Network formation →Specific death of neuronal subpopulations →Myelination

Moreover, the perturbation of these key neurodevelopmental processes (*e.g.*, decrease of synaptogenesis, decrease of neuronal network formation and function) were identified as key events (KEs) in several Adverse outcome pathways (AOPs) relevant to DNT (Bal-Price and Meek, 2017) (see Section 3).

Based on the current knowledge it can be stated that *in vitro* human neuronal models, such as those derived from hiPSCs, can recapitulate a sequence of neurodevelopmental processes starting from NPC proliferation until an advanced stage of neuronal and glial differentiation and maturation. If these processes are impaired as a result of chemical exposure, they can be assessed in a quantitative manner and serve as reliable readouts for *in vitro* DNT evaluation. Notwithstanding, further efforts should be made to upscale the throughput applicability of some measured endpoints, particularly when 3D systems are required.

### 2.1 Non-mammalian species

Evaluation of brain development using alternative (non-mammalian) species has revealed that some fundamental mechanisms underlying the development and function of the nervous system are well conserved across the phylogenic tree. Many of the basic molecular developmental processes are identical in mammals and in non-mammalian species. In the last decade, several alternative species (*e.g.*, small fish models, including *Danio rerio* (zebrafish), *Oryzias latipes* (or medaka), *etc*.) have been used as vertebrate non-mammalian models for screening neurodevelopmental toxicants (Padilla et al., 2011), especially for behavioral studies. Lower vertebrate models are relevant to DNT studies mainly for three main reasons: (1) molecular biology has revealed the basic concordance of cellular events in a wide range of small fish species to that in mammalian species, including humans; (2) the concordance has been verified with advances in genetics and pathway analyses, and (3) the size and speed of development of small fish make their use particularly ideal for medium to high throughput assays, including evaluation of behavioral changes (impossible to study using cell culture methods only). *Xenopus laevis* tadpoles have also been used to assess the neurotoxic effects of several chemicals, such as xylene and its derivatives (Gao et al., 2016) and valproate (James et al., 2015), as well as to study a variety of neurodevelopmental disorders (Pratt and Khakhalin, 2013). Among these species, due to its small size and transparency during embryogenesis, the most investigated model is the zebrafish embryo, which is considered as a non-mammalian medium-to-high throughput model mainly used for behavioral tests, as an alternative to traditional *in vivo* DNT screening (Noyes et al., 2015; Eum et al., 2016).

### 2.2 Quantitative structure-activity relationships (QSARs) approaches

According to a 2010 JRC report (Lapenna et al., 2010), there are only a few QSAR studies that have focused on the effects of chemicals on the central and peripheral nervous systems (CNS and PNS), in some cases through the modeling of *in vivo* toxicity. For example, Crofton (1996) described a SAR study of 14 different triazole fungicides which cause hyperactivity in rats (Crofton, 1996). A QSAR for PCB neurotoxicity, based on data for 28 ortho-substituted PCBs, and building on earlier work (Nevalainen et al., 1994), revealed a relationship between electronic descriptors (ELUMO, EHOMO, the ELUMO·EHOMO gap, and molecular polarizability) and the binding affinity of PCBs to the aryl hydrocarbon (Ah) receptor (Pessah et al., 2006, 2009). In particular, impairment of the developing nervous system by PCBs has been linked to their ability to alter the spatial and temporal fidelity of Ca^2+^ signaling in muscle and nerve cells through one or more receptor-mediated processes (Pessah et al., 2009). Prediction of organophosphorus acetylcholinesterase inhibition has been evaluated using 3D QSAR methods (El Yazal et al., 2001). Multivariate toxicity profiles and QSAR modeling of 21 non-dioxin-like PCBs has been also determined (Stenberg et al., 2011) based on 17 different *in vitro* screening assays on specific endpoints related to neurotoxicity.

## 3 Adverse outcome pathway concept as an underlying framework for developing *in vitro* DNT testing strategies

The Adverse Outcome Pathway (AOP) concept facilitates the application of mechanistic knowledge of toxicity pathways (*i.e.*, physiological signaling pathways perturbed upon chemical exposure) into regulatory decisions. The AOP concept describes a sequence of measurable key events (KEs) triggered by an initial interaction between a chemical and a biological target(s) (molecular initiating event, MIE). This cascade of KEs finally results in an adverse outcome (AO) (Ankley et al., 2010; Bal-Price et al., 2015b) which should be of regulatory relevance and that has traditionally been measured in mammalian toxicity studies *in vivo*. Intermediate KEs can represent pathways of toxicity at different biological levels (cellular, tissue and organ) and must be empirically observable and measurable. Empirical evidence should be based on relevant data described in the literature or studies specifically designed for the purpose of AOP development. AOPs could be useful for both the development of relevant and predictive *in vitro* test methods, as well as the identification of knowledge gaps and challenges in extrapolation of both data and models between species.

Due to the complexity of the CNS, development of AOPs relevant to DNT is challenging (Bal-Price et al., 2015b). A major concern is a general lack of understanding of the MIEs that are causally responsible for triggering KEs leading to a linear cascade of events, up to the AO observed in humans. The existing DNT AOPs are at different stages of development and, interestingly enough, most of them define cognitive impairment/learning and memory deficits in children as an AO (Bal-Price and Meek, 2017), which is of regulatory relevance. Therefore, the KEs identified in these AOPs could serve as anchors for a battery of *in vitro* assays suitable to develop a testing strategy for detecting developmental neurotoxicants with potential to cause cognitive impairment in children. Such a battery of tests that relies on mechanistic information derived from AOPs would increase scientific confidence in their use, facilitating a paradigm shift towards a mechanistically-driven hazard identification and characterization (EFSA, 2017; OECD, 2017a) and possibly risk assessment.

Taking into consideration the possible multiple MIEs leading to the same AO (*e.g.*, learning and memory impairment) and the variety of potential pathways involved, networks of AOPs should be developed, even though this will take time (Bal-Price and Meek, 2017). Indeed, an approach based on individual AOPs (assays anchored to KEs) present the limitation of being able to identify only a small number of positive “hits” (developmental neurotoxicants) eliciting toxicity through the specific AOP(s). Therefore, it has been proposed to identify “Converging Key Events” that are common to many individual AOPs (Bal-Price et al., 2015b; Bal-Price and Meek, 2017). Following this recommendation, *i.e.*, building network(s) of the existing individual AOPs relevant to DNT and determining the common KEs within such network(s), may facilitate the selection of the most critical *in vitro* assays suitable to identify a number of developmental neurotoxicants targeting various signaling pathways and resulting in the same AO, even if toxicity would be triggered by different MIEs. However, it has to be pointed out that while such an approach is suitable for screening purposes, it is not applicable for the development of QSAR models aimed at identifying chemicals triggering a specific MIE, in light of the fact that common KEs are triggered by various MIEs.

The AOP development requires description of the mechanistic, causative key event relationships (KERs) between the MIE, the KEs and the AO. If KERs are supported by a strong weight of evidence, *in vitro* assays anchored to these KEs would represent high scientific confidence in the relevance of the KE to the AO and should be used as an important component of an IATA for an initial chemical screening to identify those chemicals with DNT potential. Understanding the likelihood of effects (*e.g.*, initiation of a toxicity pathway) occurring at lower, cellular levels of biological complexity through *e.g.*, *in vitro* testing or (Q)SAR, can help to inform whether testing at higher levels of biological organisation (*i.e.*, *in vivo*) is warranted (OECD, 2016).

## 4 Development of IATA-driven by AOPs and key neurodevelopmental processes for different regulatory purposes

The development of mechanistically-informed IATA for identification of chemicals with DNT potential should be based on multiple sources of information (non-testing methods, *in vitro* approaches, *in vivo* animal and human data), delivering assessments for different regulatory purposes (*e.g.*, screening, hazard identification and characterization or risk assessment). The increasing availability of AOPs relevant to DNT will also increase scientific confidence in the use of mechanistic knowledge (AOP-informed IATA), supported by empirical data described in causative KERs. The selected *in vitro* assays included in an IATA should be anchored to MIEs and the selected set of KEs at the cellular or tissue level described in the existing DNT relevant AOPs (Bal-Price et al., 2015b; Bal-Price and Meek, 2017; OECD, 2017a), and used in a flexible combination (fit-for-purpose). Additionally, *in vitro* assays that allow an evaluation of the key neurodevelopmental processes specific for brain development but not yet described in AOPs, such as cell proliferation, migration, differentiation, *etc.*, should also be incorporated. Furthermore, mechanistic information on impairment of pathways known to be involved in these fundamental neurodevelopmental processes (reviewed in (Fritsche, 2017)), including those controlling neural precursor cell proliferation (*e.g.*, BDNF, ERK, CREB, RTK-PI3K, AKT), radial glia proliferation (*e.g.*, miRNA-17-92), migration (*e.g.*, MAP kinase, BDNF-TrkB), oligodendrocytes differentiation and myelin formation (*e.g.*, secretases, AKT-1, Nectin-like proteins, Notch, thyroid hormones, TH), neuronal differentiation (*e.g.*, mTOR, BDNF, ERK, CREB, TH, PKC) or neuronal network formation (*e.g.*, phosphoinositide metabolism, TH, BDNF/TrkB, CREB). It strongly indicates that if these pathways are sufficiently perturbed upon exposure to a chemical, leading consequentially to DNT effects. Therefore, together with KEs identified in the relevant DNT AOPs (Bal-Price and Meek, 2017), toxicity pathways analysis should also serve as anchors for DNT *in vitro* assays guiding the IATA development. A similar approach was also recommended during the OECD/EFSA DNT workshop (October 2016) (Fritsche, 2017; Fritsche et al., 2017).

### 4.1 Key considerations on DNT IATA development for an initial chemical screening and prioritization

Considering the information requirements for DNT evaluation within the existing regulations in the EU and USA, it is impossible at this time to replace animal testing with alternative *in vitro* methods. Therefore, efforts should be directed towards supporting the data derived from current *in vivo* testing following OECD TG 426, by incorporating a battery of *in vitro* methods as the first step. This would allow more targeted *in vivo* testing, improving the outcome of such studies. The proposed battery of *in vitro* DNT assays (preferably those based on human models) could be incorporated into DNT IATA designed for chemical screening and prioritization that is urgently needed, taking into consideration that so far only a few chemicals have been identified as DNT compounds.

**Fig. 2:**
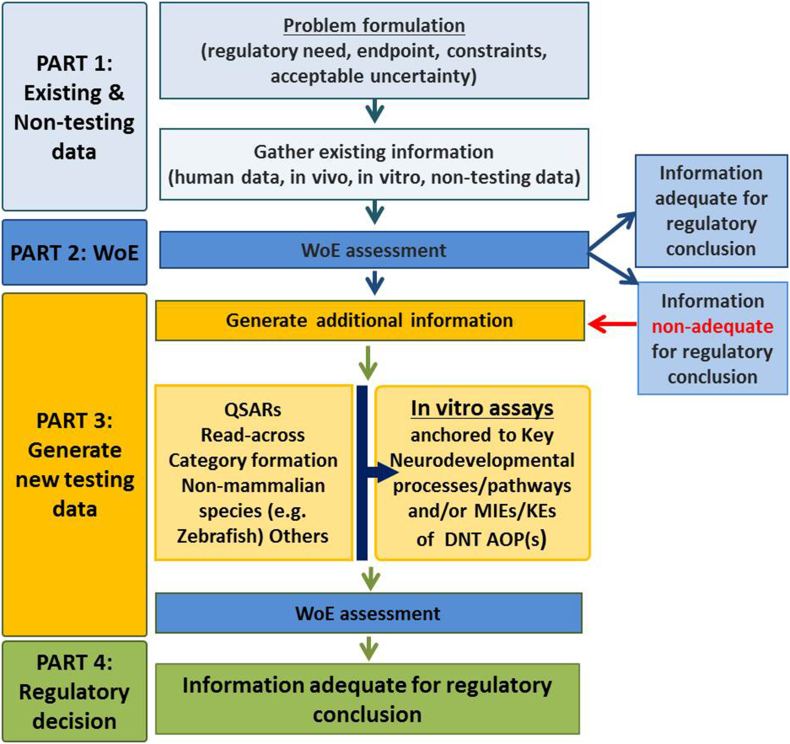
General outline of an Integrated Approach to Testing and Assessment (IATA) which integrates all available sources of existing information (human data, *in vivo, in vitro* and non-testing data) (modified from (OECD, 2016). Such an IATA can guide the targeted generation of new data based on *in vitro* DNT assays and, if required, can be combined with *in silico* approaches. Other abbreviations: WoE, weight of evidence; QSAR, quantitative structure–activity relationship; MIE, molecular initiating event; KE, key event; DNT, developmental neurotoxicity; AOP, adverse outcome pathway.

The IATA should integrate multiple sources of existing information (human data, *in vivo, in vitro* and non-testing data) and guide the targeted generation of new data, if needed (Fig. 2). If further testing is required, then, as discussed above, the battery of *in vitro* DNT tests that permit evaluations of key neurodevelopmental pathways/processes and KEs identified in the relevant AOPs, combined with non-testing methods could be incorporated in the general DNT IATA (Fig. 2) for chemical screening and prioritization purposes. A recent EFSA/OECD workshop concluded that the proposed battery based on *in vitro* DNT assays anchored to key neurodevelopmental processes and some KEs identified in the existing DNT AOPs (Bal-Price and Meek, 2017) are ready to be used for screening and prioritization purposes (Fritsche et al., 2017, EFSA, 2017; OECD, 2017a). Indeed, *in vitro* assays for cell proliferation (*e.g.*, (Mundy et al., 2010; Radio et al., 2015)), migration (*e.g.*, (Nyffeler et al., 2017)), neurite outgrowth (*e.g.*, (Harrill et al., 2013, 2015)), synaptogenesis and neuronal network formation and function (*e.g.*, by using commercially available kits based on high content image analysis or MEA measurements) (Vassallo et al., 2017) are well established based on chemical testing and can be used in a battery that would allow screening and prioritization of chemicals for their DNT properties.

Data produced from IATA will require different levels of scientific confidence and different levels of acceptable uncertainty depending on the regulatory purpose. Indeed, for screening and prioritization purposes a greater level of uncertainty could be tolerated in comparison to hazard identification and characterization, where higher levels of reliability, certainty and assay validation will be required.

Recently, a (semi)-quantitative analysis has been performed to evaluate the existing *in vitro* DNT assays according to defined readiness criteria taking into consideration different regulatory purposes (*e.g.*, prioritization/screening, hazard and risk assessment) (Bal-Price and Meek, 2017). The scoring results suggested that several assays reached high readiness levels, whereas others, such as oligodendrocytes and microglia differentiation and maturation, myelin formation, neurotransmitter release, receptor binding, and ion channels function (indicated in Fig. 1 by a red hand symbol), are not ready yet and need further optimization through chemical testing especially when performed using hiPSC-derived mixed neuronal glial cultures (Hofrichter et al., 2017).

Depending on the purpose and the substance or mixture to be evaluated, fit-for-purpose IATA may require different sets of DNT *in vitro* assays in combination with additional alternative tools, such as QSAR, *in silico* modeling and possibly non-mammalian models (*e.g.*, zebrafish), suitable for behavioral observations. Therefore, different IATA solutions may be possible depending on the chemical(s) under investigation, the regulatory purpose and context (*e.g.*, supplementing *in vivo* testing with mechanistic information, chemical screening for prioritization, hazard characterization or risk assessment).

### 4.2 Targeted *in vivo* DNT testing based on *in vitro* DNT data

Fit-for-purpose IATA could be incorporated into regulatory DNT evaluation, when they are used for hazard identification and characterization of a chemical substance, as the first tier approach before any *in vivo* testing takes place according to OECD TG 426 or TG 443.

Recently, the EFSA Panel on Plant Protection Products and their Residues (PPR Panel) in the scientific opinion on the DNT potential of the neonicotinoid insecticides acetamiprid and imidacloprid (EFSA, 2013a) commented on the current OECD TG 426 stating that "*DNT guidelines are complex, time consuming, costly and not suitable for routine testing of high numbers of chemicals. Some concerns in terms of feasibility and animal welfare have been raised in the scientific literature. Although the protocol of the guidelines is well designed and covers a broad window of exposure, the critical phase for some effects might be missed and not all effects would be found. Furthermore, the interpretation of results is difficult because of knowledge gaps concerning normal brain development on the functional, structural and molecular levels, thus complicating risk assessment of compounds* (Beronius et al., 2013). *A number of issues related to the interpretation of DNT studies have been raised such as excessive variability that may mask treatment-related effects."*

A review of the performance of *in vivo* DNT testing according to the OECD TG 426 has been also performed by scientists and regulatory bodies (Claudio et al., 2000; Makris et al., 2009). It is stated by Makris et al. (2009) that the OECD DNT guideline represents the best available science for assessing the potential for DNT in human health risk assessment, and data generated with this protocol are relevant and reliable for the assessment of these endpoints. The reproducibility, reliability, and sensitivity of these methods have been demonstrated, using a wide variety of test substances, in accordance with OECD guidance (OECD, 2005) on the validation and international acceptance of new or updated test methods for hazard characterization and multiple independent, expert scientific peer reviews affirm these conclusions.

However, evaluation of OECD TG 426 performed by Claudio et al. (2000). (Claudio et al., 2000) points out that this TG is deficient in several respects, including:

➢ It is not always triggered appropriately within the current tiered system for testing;
➢ It does not expose developing animals during all critical periods of vulnerability;
➢ It does not assess effects that may become evident later in life;
➢ It does not include methodology for consideration of pharmacokinetic variables;
➢ Methodology for assessment of neurobehavioral, neuropathological, and morphometry is highly variable and prone to subjectivity;
➢ Testing of neurochemical changes is limited and not always required.

Deficiencies in the testing methodology for developmental neurotoxicants represent a significant gap and increase the uncertainty in the establishment of safe levels of exposure to developing individuals. At the same time, since this entirely based *in vivo* guideline is very resource intensive in terms of animals, time and overall cost (Rovida and Hartung, 2009; Tsuji and Crofton, 2012), it is rarely used, resulting in a small amount of chemicals being tested for their DNT potential. This highlights the urgent need to develop IATA for screening and prioritization that can more rapidly and cost-effectively evaluate thousands of chemicals (without safety data) for their potential to cause DNT (Bal-Price et al., 2015a; Fritsche et al., 2017). Based on the IATA screening as first tier, further *in vivo* testing (if necessary) can be performed only for well targeted experiments, supported by the mechanistic information produced by a battery of *in vitro* DNT test methods.

## 5 How *in vitro* mechanistic information could support evaluation of chemical-induced DNT for different regulatory purposes

### 5.1 Hazard identification and characterization of environmental chemicals, including pesticides

Based on epidemiological studies, a link between neurodevelopmental impairment and exposure to different classes of environmental chemicals (heavy metals, POPs, *etc.*) (Grandjean and Landrigan, 2006), including pesticides, is well established (Evans et al., 2015). Pesticides are of particular importance as some of them are designed to target the nervous system function of insect pests. Because of the similarity of neurochemical processes across taxa, those pesticides are likely to be neurotoxic to humans. Therefore, concerns have been raised that the developing brain may be particularly vulnerable to adverse effects of neurotoxic pesticides (Bjorling-Poulsen et al., 2008).

Based on experimental studies, the existing data suggest that many different classes of pesticides, currently used in Europe – including organophosphates, carbamates, pyrethroids, ethylenebisdithiocarbamates, and chlorophenoxy herbicides – can cause neurodevelopmental toxicity and often adverse effects on brain development can be severe and irreversible (Grandjean and Landrigan, 2006; London et al., 2012). Therefore, this class of regulated chemicals should be recognized as a priority for evaluating DNT potential using different sources of information, including mechanistic *in vitro* data. The EFSA PPR Panel, in the scientific opinion on the DNT potential of the neonicotinoid insecticides acetamiprid and imidacloprid (EFSA, 2013a), recommended that "*in vitro assays may be regarded as complementary to animal testing because they may provide better understanding of the cellular/molecular mechanisms involved in developmental neurotoxicity. As such, in vitro tests could be incorporated into a DNT testing strategy to obtain mechanistic information or for purposes of screening/prioritisation*."

Following this recommendation, incorporation of supplementary information delivered from DNT *in vitro* mechanistic studies and other alternative approaches (*e.g.*, QSAR, read across) would increase weight of evidence when combined with DNT *in vivo* testing where results may often be equivocal or open to different interpretations with respect to whether or not a chemical has the capacity to cause DNT effects and, if so, by what mechanisms. This can be achieved by using a battery of *in vitro* assays which permit evaluation of a range of human key pathways that mediate DNT effects, critical neurodevelopmental processes at different developmental time points (exposure windows) and KEs identified in the existing AOPs relevant to DNT (Table 1A in (Bal-Price and Meek, 2017)), preferably by using human models derived from hiPSCs, rather than rodent test systems to avoid interspecies differences (Fritsche et al., 2015).

### 5.2 Biological groupings of chemicals

The *in vitro* DNT mechanistic information could be used as a basis for grouping of chemicals according to their biological activity and common mechanisms of toxicity or modes of action. Currently, some chemicals, including pesticides, are already grouped according to their mode of action, such as pyrethroids (binding to voltage-gated sodium channels), rotenoids (inhibiting electron transfer from iron-sulphur centres in complex I to ubiquinone), and nicotinoids (binding to nicotinic acetylcholine receptors (nAChRs) and mimicking the action of acetylcholine by opening the ion channels, which allow the entry of Na^+^ and Ca^2+^ into cells). This type of pesticide classification could be further refined based on mechanistic *in vitro* data. QSAR analysis would permit further sub-grouping of these chemicals according to their structure, as it has been done for instance for organochlorines, organophosphates or carbamates.

Furthermore, chemicals could be grouped (despite their differences in chemical structure), according to their biological activity, *i.e.* the capacity to trigger an impairment of the same neurodevelopmental process or the same MIE or KE. To facilitate biological grouping of chemicals, data could be generated by investigating the effects of chemicals at the molecular and cellular level using *in vitro* assays anchored to the MIEs and KEs identified in the relevant DNT AOPs (Bal-Price et al., 2015b; Bal-Price and Meek, 2017), preferably those amenable to high throughput screening (HTS), permitting testing of larger number of chemicals. This is based on the current available knowledge that nervous system development will be impaired when these key neurodevelopmental processes are sufficiently disturbed (Lein et al., 2005; Smirnova et al., 2014).

Some of these assays (*e.g.* neurite outgrowth) are already automated by HTS, permitting a quantitative evaluation using a range of different *in vitro* cell models (Breier et al., 2008; Mundy et al., 2010; Harrill et al., 2011; Druwe et al., 2016; Ryan et al., 2016). Biomarkers of differentiation processes have also been studied using primary rodent cultures and human NPCs (Kuegler et al., 2010) based on gene (Hogberg et al., 2009, 2010) and protein expression (Mundy et al., 2008), metabolomics (OMICS) analysis (Schultz et al., 2015) and measurements of neuronal electrical activity under the exposure to different classes of chemicals including pesticides (Vassallo et al., 2017). However, some of the assays are still low-throughput (cell migration, glial and neuronal differentiation and maturation, neuro-transmitters release, receptors and ion channels function, *etc.*) and a key issue for future research is to scale-up these test methods to medium or high throughput level to increase a speed of testing.

### 5.3 Assessment of combined exposures to multiple chemicals (mixture risk assessment, MRA)

Studies describing single chemical-induced toxicity do not reliably reproduce real life exposure scenarios, since foetuses, babies and children are indisputably co-exposed to more than one chemical at a time, as demonstrated in several epidemiological studies assessing the presence of several environmental chemicals in human biological samples, such as breast milk (Schlumpf et al., 2010) and cord blood (de Cock et al., 2014). Breast milk and cord blood samples have been found to contain chemicals regulated as pesticides, along with those regulated as cosmetics (including UV filters parabens, phthalates), together with persistent organic pollutants (POPs) including polychlorinated biphenyls (PCBs) (Schlumpf et al., 2010), confirming that babies are simultaneously exposed to multiple chemicals. Therefore, there is an urgent need to initiate MRA in relation to DNT. However, from a regulatory standpoint, this represents a challenging task, as chemicals that are known to trigger specific DNT effects belong to different chemical classes (*e.g.*, organic solvents, metals) or use categories (*e.g.*, pharmaceutical drugs, industrial chemicals or pesticides). Approximately 218 chemicals have been identified as neurotoxicants, of which 27 are metals or inorganic compounds, 41 are organic solvents, 48 are other organic substances and 102 are pesticides (Grandjean and Landrigan, 2014). In a more recent study by Maffini and Neltner (2015), more than 300 chemicals were identified as potential DNT chemicals. These compounds belong to various regulatory use categories related to food quality, such as pesticides, food contact material and food additives including flavourings, colourings and preservatives. These examples illustrate that common, similar or related toxic effects triggered by various chemicals may be differently regulated, and that combined effects of these chemicals across different regulatory domains are not currently considered (Evans et al., 2016). For this reason, current European chemical regulations operating on a chemical-by-chemical basis (*i.e.*, in silos) may result to be too restrictive and/or poorly flexible in cases of combined exposure to multiple chemicals (Evans et al., 2016). Evans and colleagues suggested that, instead of limiting MRA to chemicals belonging to one or another regulatory silo, a more flexible and horizontal approach, spanning different regulatory silos, should be considered to fully capture human risk (Evans et al., 2016).

At the same time it is well documented in the existing literature that “mixture effects” can be greater than effects triggered by the most potent single chemical, and the combined exposure effects may be additive or in some cases even synergistic (Kortenkamp et al., 2009, 2012; Kienzler et al., 2016). Different approaches for cumulative risk assessments have been discussed in two EFSA Scientific opinions (EFSA, 2013b,c). The EFSA PPR panel suggested that assessment of cumulative risk could be performed considering chemicals acting through similar mode of action (MoA) *versus* those acting through dissimilar MoA, but all leading to the same AO (*e.g.*, cognitive impairment and/or mental retardation in children), supporting the concept of dose addition (EFSA, 2013b). The EU regulation on maximum residue levels (MRLs) in food stipulates that decisions on MRLs should take into account cumulative effects of pesticides when the methods to assess such effects become available.

In this context, a battery of *in vitro* assays anchored to common KEs of AOPs relevant to DNT (*e.g.*, reduced synaptogenesis, reduced neurite outgrowth, reduction of BDNF, measured both at gene and protein level) but triggered by various MIEs could be a suitable approach to assess the effects of chemical mixtures to evaluate possible additive, synergistic and antagonistic effects.

### 5.4 Supporting identification of potential triggers for DNT testing by *in vitro* studies

As mentioned above, DNT testing is not a mandatory requirement in EU or USA legislation and is only performed when triggered. A DNT study can be conducted as a separate study, incorporated into a reproductive toxicity and/or adult neurotoxicity study (*e.g.*, TG 443 (OECD, 2011), 416 (OECD, 2001b), 424 (OECD, 1997)), or added onto a prenatal developmental toxicity study (*e.g.*, TG 414 (OECD, 2001a)). For example, the REACH technical guidance document on information requirements makes similar recommendations stating that “*in exceptional cases when relevant triggers are met, testing for developmental neurotoxicity effects should be considered*” (ECHA, 2015). The existing criteria for triggering DNT testing are based on systemic *in vivo* study data (acute or repeated-dose toxicity studies) in adult rodents. Relevant triggers (ECHA, 2015) are defined if the substance has been shown to:

(1) cause structural abnormalities of the central nervous system,
(2) induce clear signs of behavioral impairments or functional adverse effects on the nervous system in adult studies,
(3) have structure-activity relationships similar to a known neurotoxic chemical,
(4) have a mode of action that has been closely linked to neurotoxic or developmental neurotoxicity effects (*e.g.*, cholinesterase inhibition or thyroid effects).

However, while these triggers are biomarkers of neurotoxicity, they may not be specific for brain development. Furthermore, they are observed in adult animals that are not always a relevant model for the evaluation of impaired processes and pathways specific for brain development. Complexity of the processes specific for the developing brain is very different from those taking place in the adult (mature) brain. Therefore, observations carried out in adult animals and in the adult brain cannot be considered as proxies of processes specific of the brain under development, including (a) neurogenesis (*i.e.*, neurons formation), (b) migration of neurons toward specific brain areas, (c) myelination (*i.e.*, coating of the axons with myelin sheets), (d) pruning (*i.e.*, removal of unnecessary connections), (e) synaptogenesis, and (f) neuronal networks formation and function. Such processes are very dynamic and restricted only to certain windows of brain development and do not occur in an adult brain. As discussed earlier, these key neurodevelopmental processes (and many others) (Bal-Price et al., 2017) can be reconstructed using hiPSC-derived neuronal models (Emdad et al., 2012; Lancaster et al., 2013; Scott et al., 2013; Pistollato et al., 2014; Pamies et al., 2017b).

By using human *in vitro* neuronal/glial cell cultures, it has been shown that exposure to the same chemical at different stages of brain development results in different levels of toxicity. For instance, NPC formation is much more sensitive to methylmercury at non-cytotoxic concentrations than their differentiated, more mature counterpart (Stummann et al., 2009). Although some of these processes, *e.g.*, neurogenesis or synaptogenesis originating from adult stem cells, under certain circumstances, may also occasionally take place in the adult brain, under certain circumstances, the dynamics, speed and scale are incomparable to those processes occurring in the developing brain (embryo, foetus, babies). Considering that the developing brain is known to be much more vulnerable than the adult brain to chemical exposure, potential triggers for DNT studies based on observations in adult animals are not likely not to be reliable or accurate for making confident decisions regarding whether or not DNT studies are required. This may represent one of the reasons why DNT studies are so rarely performed.

Here, we propose to redefine in a more informed, mechanistically-based manner the criteria for triggering DNT testing based on *in vitro* studies which should support the current *in vivo* triggers for DNT testing. A battery of *in vitro* test methods would permit evaluation of key processes specific to the brain at different developmental stages, using human cell-based models derived from hiPSCs. Such an *in vitro* approach would permit to apply different exposure scenarios, targeting different windows of brain development, that are difficult to study in the adult brain.

Including triggers based on human *in vitro* DNT studies would increase the probability of flagging chemicals with potential to cause DNT effects, whilst allowing gathering detailed mechanistic information regarding which specific neurodevelopmental processes and/or signaling pathways are affected and at what stage of brain development (Fritsche et al., 2017). If these signaling pathways or key neurodevelopmental processes would be affected by a chemical at the concentrations relevant to human exposure based on PBPK modeling, providing information on internal exposures at the target site, perhaps such information could be sufficient to take regulatory decision, avoiding further *in vivo* studies. Depending on the regulatory context (*e.g.*, risk assessment), such information could also serve as a potential trigger for further *in vitro* or, if necessary, *in vivo* DNT testing. This approach could be of particular importance in the process of “substance evaluation” under REACH, relevant to the identification of a SVHC (Substance of Very High Concern). Currently, DNT evaluation may be triggered based on the symptoms observed in systemic studies where, depending on the tonnage level, a 28-day and/or a 90-day repeated-dose toxicity evaluation is performed (*e.g.* OECD TG 407, 408). In case of chemicals produced or imported with volumes of over 10 t/y, repeated dose 28-day oral toxicity testing (OECD TG 407) is required and a combined repeated dose toxicity study with the reproduction/developmental toxicity screening test (OECD TG 422 (OECD, 1996)) is recommended. At volumes over 100 t/y, a sub-chronic toxicity study (90-day) toxicity study (OECD TG 408) is required, which can be waived under certain circumstances. Importantly, incorporating supplementary *in vitro* identified triggers for DNT testing would be in compliance with the REACH regulation.

## 6 Discussion

New approaches in toxicology including the AOP and IATA concepts, the use of *in vitro* human stem cell-derived neuronal models, QSARs and read across used in an integrated manner, may pave the way to a more efficient and predictive assessment of DNT, solving various regulatory challenges.

In the recent DNT workshop that was co-organised by the OECD and EFSA, there was strong support for the development of *in vitro* battery of DNT relevant methods in order to screen a high number of chemicals for their DNT potential (EFSA, 2017; OECD, 2017a). The battery of methods should be composed of robust, reliable and standardized *in vitro* assays, relevant for the assessment of human toxicity to support a tiered, cost-effective chemical screening, hazard identification and characterization, within a risk assessment context.

Towards this goal, the strategy outlined here would be to produce data in a much more targeted manner, focusing on DNT-specific endpoints, using *in vitro* methods. Therefore, IATA has been proposed as a practical solution to provide testing strategies composed of *in vitro* assays anchored to KEs identified in DNT-related AOPs and key neurodevelopmental processes. In parallel, other AOPs relevant to DNT should be further developed, as they will expand mechanistic knowledge on the causal links between MIEs, KEs and AOs of regulatory concern, providing the biological context for the *in vitro* assays, and facilitating the development of AOPs network and AOP-informed IATA for regulatory decision-making.

Currently, the main task is to establish performance standards and readiness criteria for the evaluation of individual *in vitro* DNT assays to determine which assays are ready for which regulatory purposes. Such knowledge will lead to development of guidance on how to build a testing strategies consisting of *in vitro* methods combined (if necessary/where relevant) with methods based on non-mammalian organisms and/or non-testing methods.

In addition, it will be critical to be able to define threshold(s) for key events, allowing discriminating between changes observed in *in vitro* studies as adaptive processes normally found in biological systems *in vivo*, from those that are predictive of adverse outcomes. Coupling the adverse or adaptive nature of the measured endpoints with absorption, distribution, metabolism, and excretion (ADME) data and exposure information derived from *in vitro* to *in vivo* extrapolation (IVIVE) would increase the level of confidence in the information derived from *in vitro* methods. Indeed, for the regulatory applications, data derived from *in vitro* models relevant to human biology would be greatly enhanced if coupled with models of chemical kinetics and dynamics, being more predictive for an *in vivo* exposure scenario. Moreover, a battery of *in vitro* alternative approaches should address several aspects related to quality controls, reproducibility, sensitivity, specificity, and predictive capacity of obtained *in vitro* DNT data suitable for a defined regulatory purpose. Currently available *in vitro* DNT alternative test methods could be already applied for the generation of data to prioritize chemicals for further testing. However, to generate data that would inform risk management decisions, some of these assays need further optimization and standardization through further testing with a wide range of chemicals. Recently, a list of readiness criteria has been compiled during a stakeholder workshop, and a semi-quantitative evaluation of 17 *in vitro* DNT assays has been performed (Bal-Price et al., 2017). Some of these assays have reached high scores for readiness levels (*e.g.*, neural precursors proliferation, migration, differentiation), whilst some others need further optimization (*e.g.*, myelin formation). Based on this information and experimentally generated new *in vitro* data, an OECD Guidance Document on available *in vitro* DNT test methods, used alone or in combination, within the context of an IATA, will be developed for various regulatory purposes. Such a Guidance Document on a suitable *in vitro* DNT battery of assays, principles of developing DNT IATA for various regulatory applications, data interpretation and building prediction models, has been included in the OECD Work programme, and will be developed in collaboration with EFSA and DNT experts from OECD member countries in the near future.
